# Brain iron load and neuroaxonal vulnerability in adult attention‐deficit hyperactivity disorder

**DOI:** 10.1111/pcn.13806

**Published:** 2025-02-27

**Authors:** Jatta Berberat, Sonja M Kagerer, Claudia Späni, Jun Hua, Francesco Bavato, Philipp Gruber, Peter CM van Zijl, Nader Perroud, Xu Li, Philipp Stämpfli, Erich Seifritz, Karl‐Olof Lövblad, Boris B Quednow, Paul G Unschuld

**Affiliations:** ^1^ Division of Geriatric Psychiatry University Hospitals of Geneva (HUG) Thônex Switzerland; ^2^ Department of Psychiatry University of Geneva (UniGE) Geneva Switzerland; ^3^ Institute of Neuroradiology, Kantonsspital Aarau Aarau Switzerland; ^4^ Department of Geriatric Psychiatry and Psychotherapy University Hospital of Psychiatry Zurich, University of Zurich; ^5^ Institute for Regenerative Medicine (IREM), University of Zurich Schlieren Switzerland; ^6^ Neurosection, Division of MRI Research, Department of Radiology Johns Hopkins University School of Medicine Baltimore Maryland USA; ^7^ F.M. Kirby Research Center for Functional Brain Imaging Kennedy Krieger Institute Baltimore Maryland USA; ^8^ Department of Adult Psychiatry and Psychotherapy University Hospital of Psychiatry Zurich Zurich Switzerland; ^9^ Neuroscience Center Zurich, University of Zurich and Swiss Federal Institute of Technology Zurich Switzerland; ^10^ Division of Diagnostic and Interventional Neuroradiology Geneva University Hospitals and Faculty of Medicine of Geneva Geneva Switzerland

**Keywords:** ADHD, dementia, iron, MRI, NfL

## Abstract

**Aim:**

Adult attention deficit hyperactivity disorder (ADHD) may be associated with an increased risk of dementia in old age. Here, we investigated the liability for neurodegenerative brain disease in adult ADHD, possibly reflected by increased brain iron content and associated neuroaxonal vulnerability.

**Methods:**

Thirty‐two adults with ADHD (35 ± 10 years) and 29 age‐ and sex‐matched controls (32 ± 12 years) underwent magnetic resonance imaging (MRI), standardized psychometric testing and assessment of lifestyle factors. Quantitative susceptibility mapping (QSM) was used to assess magnetic abnormalities indicating local alterations of iron deposition in the brain. By calculating QSM‐maps, local iron deposition was tested for statistically significant differences between ADHD and healthy controls. Plasma neurofilament light chain (NfL) levels were measured as an indicator of neuroaxonal integrity by using a fourth‐generation ELLA immunoassay.

**Results:**

Brain iron content differed in persons with ADHD, with strongest effects observable in the right precentral cortex (healthy controls: 0.0033 ± 0.0017ppm; ADHD: 0.0048 ± 0.0016ppm; *t*(59) = 3.56, *P <* 0.001). Moreover, right precentral cortex iron in persons with ADHD was associated with increased blood NfL levels (*F*(1.57) = 13.2, *P =* 0.001, *r* ^*2*^ = 0.19).

**Conclusion:**

Our results indicate altered regional iron content in the brains of adults with ADHD. The observed association between increased precentral magnetic susceptibility and increased NfL suggests a connection between local excess of brain iron and neuroaxonal damage in ADHD. Given the limited sample size of the current study and the naturalistic medication plan, further longitudinal studies are needed to establish whether altered brain iron distribution in adults with ADHD may be associated with an increased risk of dementia at old age.

Attention‐deficit hyperactivity disorder (ADHD) is a common neurodevelopmental disorder characterized by inability to sustain attention, inappropriate levels of hyperactivity, and impulsivity.^1^ ADHD symptoms usually manifest in childhood and significantly affect academic development and social interactions with a prevalence of approximately 3.5% in adults.^2^ A diagnosis of adult ADHD has been suggested to be associated with an increased risk of Alzheimer's disease and dementia in old age in several longitudinal, nationwide cohort studies.^3^, ^4^, ^5^ While causality of ADHD, Alzheimer's disease and dementia is complex, mechanisms that may link these clinically distinct disorders are unknown.^6^

The development of ADHD is influenced by a complex interaction of genetic and environmental factors.^7^, ^8^, ^9^ There is no single test to diagnose ADHD, and the diagnosis can be made in both young people and adults based on the presence of the characteristic behavioral syndrome.^6^ Adult ADHD is often associated with an increased prevalence of cardiovascular risk factors such as smoking, obesity, diabetes^10^, ^11^ and altered lipid profiles,^11^, ^12^ all of which are well established risk‐factors for age‐related cognitive decline and Alzheimer's disease.^13^, ^14^

Several studies consistently show that the aging human brain is characterized by a continuous accumulation of iron in both subcortical and neocortical areas.^15^, ^16^, ^17^ This may reflect both the involvement of iron in many neurobiological processes and the difficulty in removing excess iron from the central nervous system, possibly exacerbated by lifestyle factors such as body mass index (BMI) and smoking.^18^ Increased brain iron is a well‐known finding in the elderly, increased brain iron has been reported to be generally associated with reduced cognitive performance.^19^ Furthermore, increased brain iron is a characteristic finding in several neurodegenerative diseases including Huntington's disease,^20^, ^21^ Parkinson's disease,^22^, ^23^ and Alzheimer's disease.^20^, ^24^, ^25^, ^26^

Iron in the brain can be measured by Magnetic Resonance Imaging (MRI), using Quantitative Susceptibility Mapping (QSM), which uses the magnetization induced in tissue when placed in a magnetic field to provide a quantitative measure of the tissue susceptibility distribution.^16^, ^27^ Compared to other techniques currently used in clinical brain imaging such as susceptibility weighted imaging (SWI),^28^ QSM and R2* mapping provide improved detection and identification of iron and calcium deposition, improved characterization of pathological iron and myelin variations, and unique details of brain morphology within deep brain nuclei.^29^, ^30^ The fundamental difference between QSM and R2* mapping is that para‐ and diamagnetic inclusions have opposite effects on the magnetic susceptibility (χ) estimates obtained from QSM, but the same effect on the R2* rate.^31^ At this point, several QSM‐MRI studies have shown an association between increased tissue magnetic susceptibility and accelerated cognitive decline by demonstrating the possible detrimental effects of brain iron burden on brain tissue.^32^, ^33^ In this respect it is important to point out that these results are based on the assumption that increased susceptibility is due to increased iron content, which is generally applicable in gray matter, but has a caveat in white matter because demyelination will also increase the susceptibility.^17^, ^30^ While reduced iron distribution in the brain is a confirmed finding in children with ADHD,^34^, ^35^ information about iron distribution in the brain of adults with ADHD is limited. To our knowledge, so far, no QSM studies have been published on adult ADHD.

Neurofilament light chain protein (NfL) is a neuronal cytoskeletal protein that is highly expressed in myelinated axons of the brain. Elevated peripheral blood serum NfL is an established biomarker for neuroaxonal damage in the central nervous system (CNS).^36^ Fourth‐generation enzyme‐linked immunoassay technologies such as SiMoA or ELLA have been used to investigate NfL in various neurological and psychiatric disorders.^37^, ^38^, ^39^ Notably, elevated NfL has been observed in manifest AD,^40^, ^41^, ^42^ persons at genetic risk for AD^43^ and also in physiological brain aging.^41^, ^44^ Results from a recent study on Parkinson's disorder support increased discrimination validity when combining NfL‐blood assays and QSM neuroimaging.^45^ To our knowledge, possible CNS neuroaxonal damage in ADHD has not yet been investigated, and there are currently no published studies on altered NfL levels in adult ADHD.

The present study sought to examine the variability in brain iron levels in adults diagnosed with ADHD as a neuropathological alteration, with a potential association to the established link between adult ADHD and an increased risk of developing dementia in later life.^3^, ^4^, ^5^ To this end, individuals with a diagnosis of ADHD and healthy controls were examined using QSM‐MRI. The individual measurements of brain iron levels were then combined with plasma NfL levels, providing an indicator of present neuroaxonal vulnerability of the CNS.

## Methods

### Recruitment and characterization of the study population

The study population comprised adults with a diagnosis of ADHD (*n* = 32, age: 35 ± 10 years [range: 20–58 years], 17 females), and age‐, sex‐, as well as education‐matched healthy controls (*n* = 29, age: 33 ± 12 years [range: 19–59 years], 21 females). Participants were recruited at the Center for Psychiatric Research, Psychiatric University Hospital, Zurich, Switzerland.

Inclusion criteria for the current study were a minimum age of 18 years and consent to the study protocol, including willingness to participate in an MRI examination, psychometric‐neuropsychological testing and a blood test. For the ADHD group, additionally, a clinical diagnosis of ADHD (DSM‐5) was a mandatory requirement for study inclusion. Exclusion criteria for all participants was presence of addiction disorder, including abusive use of alcohol, recreational drugs or illegal drugs. Also, any present psychiatric disorder such as bipolar disease, recurrent unipolar depression, psychotic disorder and obsessive‐compulsive disorder were exclusion criterion for controls and patients. Further exclusion criteria were the presence of neurological brain disease, including severe traumatic brain injury, multiple sclerosis, known cerebrovascular disease and stroke, brain tumor, central nervous system infections, known disorders of iron metabolism.

Assignment to the patient group was based on the diagnosis of ADHD by the treating outpatient psychiatric specialist according to DSM‐5 criteria. 59.4% of study participants with ADHD received a medication with methylphenidate or dexamphetamine, which was not changed in the context of study participation, thus allowing for the naturalistic, observational design of our study. All participants underwent standardized psychometric exploration the Adult ADHD Self‐Report Scale, items 1–18, (ADHD‐SR) based on Rösler and Retz^46^ as well as the Wender‐Utah‐Rating‐Scale 25 item version (WURS‐k) as a self‐report on presence and severity of childhood symptoms of ADHD in adults.^47^ Lifestyle factors known to affect cerebrovascular health were documented, including smoking and body mass index (BMI). The protocol for the research project was approved by the local ethics committee (Kantonale Ethikkommission Zürich, BASEC‐Nr.: 2020–00103) and conforms to the tenets of the Declaration of Helsinki. Informed consent was obtained from all participants.

### MRI studies

Participants underwent 3T head MRI scan (Achieva, Philips Healthcare) with a 32‐channel head coil. Sagittal 3D isotropic T1 mprage sequence was performed using the following parameters: repetition time (TR) = 6.7 ms, echo time (TE) = 3.1 ms, fip angle (fa) = 9°; voxel size = 1 × 1 × 1 mm^3^, field of view (FOV) = 224 × 224 × 140 mm^3^, one average, acquisition time (TA) = 4:73min. Thereafter, T1 fast field echo (FFE) sequence was run with five unipolar echoes to obtain magnitude and phase images for QSM analysis (TR = 40 ms; TEs = 6, 12, 18, 24, 30 ms; fa = 15°; voxel size = 1 × 1 × 1 mm^3^, FOV = 224 × 224 × 140 mm^3^, 1 average, compressed sensing: 6×, TA = 4:52 min). Additionally, FLAIR sequence was performed (TR = 8000 ms; TEs = 135 ms; fa = 90°; voxel size = 1 × 1 × 1 mm^3^, 2 averages, TA = 2:67 min) and Fazekas score^48^ was determined as a measure of white matter signal abnormalities (WMSA).

### QSM‐analysis

**Fig. 1:**
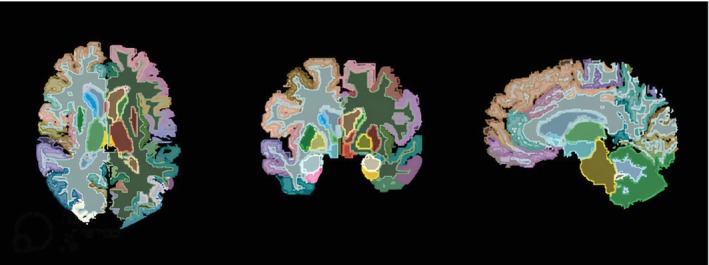
Example of the segmented ROI positions based on Freesurfer aseg‐atlas.

The QSM maps were calculated using the QSMxT framework.^49^ The phase unwrapping, background field removal and dipole inversion steps were achieved using the single‐step TGV‐QSM algorithm.^50^ QSM reconstruction was performed using a two‐pass QSM procedure.^49^ The two‐pass QSM technique and its masking strategies including motion correction are automated within the open‐source QSMxT framework. After a combined QSM image was reconstructed for each echo, a final weighted average was produced by summing the combined QSMs and dividing them by the pixel‐wise sum of the filled masks. T1‐weighted images were segmented using FreeSurfer *via* their Aseg probabilistic atlas (Fig. 1). Then, T1‐weighted anatomical images were registered to the averaged magnitude in the QSM space using the Medical Imaging NetCDF extension (minc toolkit) for referencing the whole brain (Version 1.9.18, https://bic-mni.github.io/). QSM statistics for each ROI were reported based on the ICBM152 Linear atlas (https://en.wikibooks.org/wiki/MINC/Atlases).

### Blood testing for NfL

Blood plasma samples were obtained *via* the use of EDTA tubes. Samples were centrifuged for 15 min at 2000 rpm and 20°C and subsequently stored at −80°C. NfL concentration in the plasma was measured using simple‐plex NfL assay (ProteinSimple, CA, USA) on the ELLA enzyme‐linked immunosorbent assay (ELISA) microfluidic system (BioTechne, Minneapolis, USA).

### Statistics

*QSM*: Statistical analysis was performed using SPSS statistics software version 24 (IBM Corporation, Armonk, NY, USA) and MathWorks' MATLAB 2022a data analysis software Version 9.12.0 (MathWorks Inc., Natick, MA, USA). An unpaired t‐test was used to test for statistically significant differences in normal distributed QSM values between ADHD patients and healthy volunteers. *P*‐values are indicated after controlling the false discovery rate (FDR) using the Yoav Benjamini and Yosef Hochberg approach to correct for performing 90 tests.^51^ Results are expressed as mean ± SD.

*NfL*: Mann–Whitney *U* and Spearman rho were used to test for statistically significant differences in NfL values between ADHD patients and healthy volunteers, as well as statistical association with self‐report scales and plasma NfL (pg/mL).

*Lifestyle*: ANOVA was used to examine the relationships between lifestyle factors known to be associated with impaired vascular health (BMI, age, smoking habits, alcohol) and QSM values of each ROI.

NfL *vs*. QSM: The MATLAB (fitglm.m) was used to investigate possible interactive effects of NfL (pg/mL) and iron as indicated by QSM values (ppm) with diagnostic status (ADHD *versus* controls) by generalized linear regression, and to generate the scatter plot and regression lines for NfL and QSM.

## Results

### Characteristics of the study samples

The demographic information for all the subjects and the clinical test results on all participants are summarized in Table 1. There were no statistically significant differences between the studied groups regarding lifestyle and vascular risk factors such as high body mass index (BMI) and smoking habits as well as age and sex. Two‐tailed *t*‐test of Fazekas scores did not provide evidence for significant differences in white matter signal abnormalities and cerebrovascular pathology between ADHD and healthy controls (ADHD: 0.09 ± 0.29; healthy controls: 0.12 ± 0.43; *t*(59) = 0.32; *P =* 0.75).

**Table 1:** Pooled demographic information of all the subjects (mean ± SD), lifestyle factors based on self‐reported scale

	Healthy controls (*n* = 29)	ADHD (*n* = 32)	*t*‐test (*P*‐values)
Sex (f/m)^†^	21/8	17/15	0.10
Age (years)	32.55 ± 11.94	36.22 ± 10.35	0.20
Weight (kg)	63.85 ± 8.58	68.10 ± 11.48	0.10
Height (cm)	171.63 ± 6.80	173.25 ± 8.24	0.40
BMI (kg/m^2^)	21.64 ± 2.29	22.62 ± 3.05	0.16
Education (years)	16.58 ± 2.54	16.63 ± 3.80	0.96
Nicotine consumption (×cigarettes per day)	0.62 ± 1.74	0.61 ± 1.15	0.96
Alcohol consumption (×drinks per month)	5.90 ± 6.75	5.73 ± 6.40	0.92
WURS‐k (sum 25 items)	17.66 ± 11.59	43.74 ± 13.08	<0.001
ADHD‐SR (sum items 1–18)	4.17 ± 3.96	14.63 ± 1.72	<0.001
Medication with Methylphenidate or Dexamphetamine (%)	0%	59.4%	<0.001
Fazekas Score	0.09 ± 0.29	0.12 ± 0.43	0.82

^†^ f, female; m, male.

### Local cerebral iron is increased in the right precentral gyrus of adults with ADHD, with different distributions in both groups

**Fig. 2:**
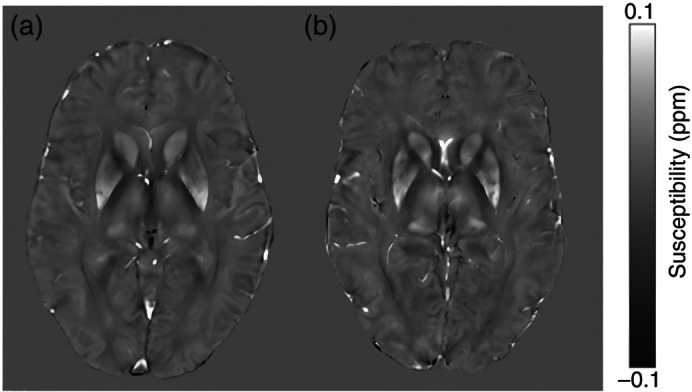
(a and b) Quantitative susceptibility maps for (a) a healthy control (42‐year‐old female) and (b) a subject with ADHD (26‐year‐old female). Increased susceptibility values are visible in iron‐rich deep gray matter structures, such as the caudate nucleus, precuneus and caudal anterior cingulate on ADHD population.

**Fig. 3:**
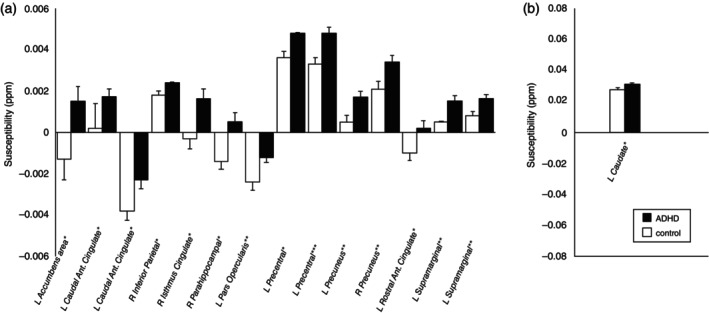
(a and b) Magnetic susceptibility values (in ppm) of the controls (white) and ADHD patients (black). Error bars represent standard error of mean (SEM). Statistically significant differences in QSM values between the controls and ADHD group were detected bilaterally in the precentral area, cerebral white matter, caudal anterior cingulate cortex, and precuneus; at left nucleus accumbens, pars opercularis, caudate, rostral anterior and isthmus cingulate cortex, right inferior parietal cortex, and parahippocampus. *(*P <* 0.05), **(*P <* 0.01), ***(*P <* 0.001) indicate statistically significant difference of QSM values between the control and ADHD groups.

Figure 2a,b shows example susceptibility maps, indicating the regional distribution of brain iron, for a healthy control and an individual with ADHD. To detect differences in mean ROI‐susceptibility between controls and ADHD patients, 90 t‐tests were performed. Resulting *P*‐values were adjusted for multiple testing by using Benjamini‐Hochberg FDR control,^51^ suggesting differences in iron levels between controls and ADHD patients in 17 brain regions: precentral cortex (bilateral), supramarginal gyrus (bilateral), cerebral white matter (bilateral), caudal anterior cingulate cortex (bilateral), precuneus (bilateral), left nucleus accumbens, left pars opercularis, left caudate, left rostral anterior and left isthmus cingulate cortex, right inferior parietal cortex and right parahippocampus (Table 2, Fig. 3a,b).

**Table 2:** Summary of the susceptibility (*χ*) data. *P*‐values are indicated in three levels of significance after controlling the false discovery rate.^51^ ANOVA was used to investigate relationships between life‐style factors and QSM values

	Healthy controls (*n* = 29)			ADHD (*n* = 32)			ANOVA
	*χ* (ppm)	*χ* (ppm)	*χ* (ppm)	*χ* (ppm)	*χ* (ppm)	*χ* (ppm)	
ROI	mean ± SD	min	max	mean ± SD	min	max	*F*(1, 41)
L Accumbens area*	−0.0013 ± 0.0055	0.0065	0.0155	0.0015 ± 0.0041	0.0015	0.0240	1.35, 0.251
L Caudate*	0.0273 ± 0.0065	−0.0117	0.0104	0.0308 ± 0.0056	−0.0066	0.0096	1.11, 0.432
L Cerebral‐WM**	−0.0030 ± 0.0011	−0.0067	−0.0019	−0.0038 ± 0.0009	−0.0076	−0.0021	1.14, 0.432
R Cerebral WM**	−0.0030 ± 0.0010	−0.0058	−0.0013	−0.0038 ± 0.0009	−0.0062	−0.0021	1.12, 0.409
L Caudal Ant. Cingulate*	0.0002 ± 0.0023	0.0019	0.0310	0.0017 ± 0.0023	0.0034	0.0401	1.12, 0.313
R Caudal Ant. Cingulate*	−0.0038 ± 0.0026	0.0101	0.0316	−0.0023 ± 0.0023	−0.0008	0.0052	0.408, 0.691
R Inferior Parietal*	0.0018 ± 0.0011	0.0113	0.0332	0.0024 ± 0.0009	−0.0044	0.0074	1.15, 0.390
R Isthmus Cingulate*	−0.0003 ± 0.0028	0.0121	0.0342	0.0016 ± 0.0028	−0.0076	−0.0011	0.81, 0.720
R Parahippocampal**	−0.0014 ± 0.0021	0.0138	0.0365	0.0005 ± 0.0026	−0.0016	0.0027	1.06, 0.463
L Pars Opercularis**	−0.0024 ± 0.0022	0.0064	0.0267	−0.0012 ± 0.0015	0.0001	0.0136	1.61, 0.140
L Precentral Cortex*	0.0036 ± 0.0017	0.0078	0.0287	0.0048 ± 0.0002	−0.0028	0.0059	1.748, 0.101
R Precentral Cortex***	0.0033 ± 0.0017	0.0156	0.0388	0.0048 ± 0.0016	−0.0047	0.0053	0.623, 0.896
L Precuneus**	0.0005 ± 0.0018	0.0081	0.0290	0.0017 ± 0.0016	−0.0036	0.0076	1.36, 0.244
R Precuneus*	0.0021 ± 0.0021	0.0158	0.0391	0.0034 ± 0.0017	−0.0020	0.0067	1.10, 0.427
L Rostral Ant. Cingulate*	−0.0010 ± 0.0019	0.0083	0.0293	0.0002 ± 0.0021	−0.0009	0.0111	1.02, 0.505
L Supramarginal Gyrus**	0.0005 ± 0.0009	0.0096	0.0309	0.0015 ± 0.0015	−0.0029	0.0047	0.93, 0.597
R Supramarginal Gyrus*	0.0008 ± 0.0011	0.0173	0.0410	0.0016 ± 0.0013	−0.0023	0.0036	1.25, 0.315

Abbreviations: Ant, anterior; L, left; R, right; SD, standard deviation; WM, white matter.

* Statistically significant difference (*P <* 0.05) of the mean qsm values between the groups.

** Statistically significant difference (*P <* 0.01) of the mean qsm values between the groups.

*** Statistically significant difference (*P <* 0.001) of the mean qsm values between the groups.

### No significant association between lifestyle factors and brain iron in ADHD or healthy controls

ANOVA‐testing for possible associations between lifestyle factors and QSM values did not reveal statistically significant effects for either of the two groups studied (Table 1). No significant correlation was found between ADHD severity (as measured by the ADHD‐SR and WURS scales) and individual brain iron distribution as indicated by regional QSM values of the 17 brain regions with differing iron level in ADHD *versus* healthy controls (Table 2).

### Right precentral cortex iron is associated with elevated blood NfL in the ADHD group

**Fig. 4:**
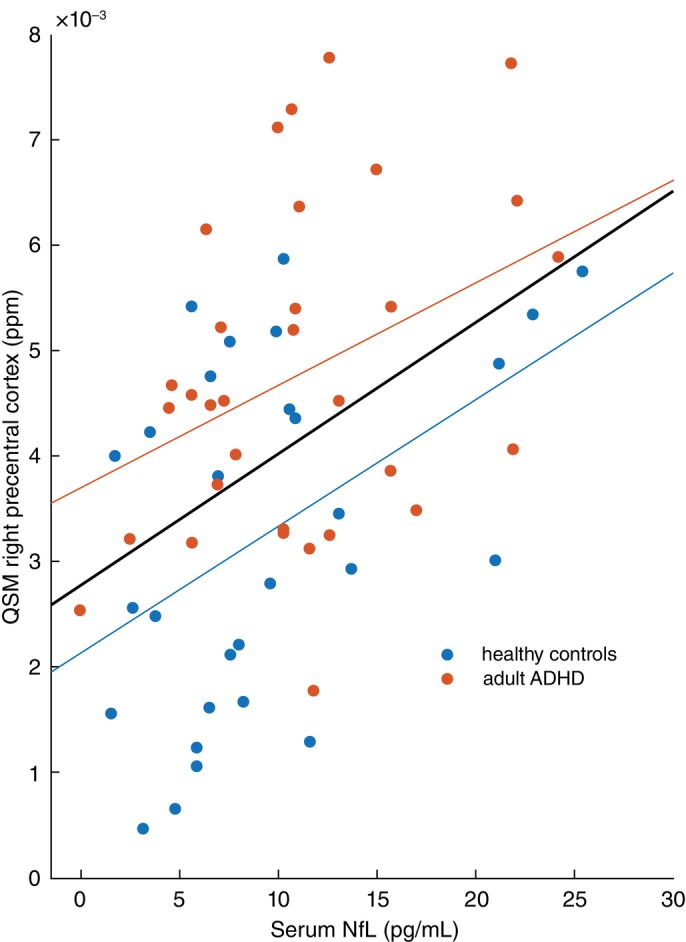
Scatterplot of QSM values of the right precentral cortex (ppm) and serum NfL (pg/mL) for healthy controls and ADHD patients. The statistical relationship is visualized by least‐squares regression lines for each group, difference of slopes = (*F*(1.57) = 13.2, *P =* 0.001).

Plasma NfL‐levels were not significantly different between participants with ADHD (median = 10.80 pg/ml, SD = 6.22 pg/mL) and controls (median = 7.58 pg/mL, SD = 6.41pg/mL), as tested by two‐sample Mann–Whitney *U* (*U* = 387, *P =* 0.139, effect size *Z* = 0.19). Using Spearman's correlation index *rho*, no significant relationships were observed between psychometric self‐rating scores WURS‐k (*r* = 0.12, *P =* 0.34), and ADHD‐SR (*r* = 0.11, *P =* 0.40) and plasma NfL levels. To assess a relationship between iron content and neuroaxonal damage, Spearman correlation coefficients were calculated for QSM and NfL in all participants, revealing a significant positive relationship between NfL (pg/mL) and QSM‐values indicating iron content of the right precentral cortex (all participants: *r*(59) = 0.41; *P =* 0.001), with a significantly higher intercept (constant) in the ADHD group, indicating higher iron in the precentral gyrus (Fig. 4). To investigate whether NfL and precentral iron were isolated or correlated in their association with ADHD, we used a Chi^2^‐statistic *vs*. constant model, assuming a binomial distribution to build the generalized linear regression model with 60 observations. Serum NfL and QSM values indicating local iron in the right precentral cortex, were interactively associated with ADHD status (*F*(1.57) = 13.2, *P =* 0.001). Binomial logistic regression to calculate coefficients of determination indicated a 19% predictability of ADHD status when right precentral cortex iron load (as estimated by QSM ppm) and blood NfL were included in the model (Nagelkerke *r* ^2^ = 0.19). The correlation matrix of the logistic regression test statistic demonstrated that the right precentral cortex iron contributed significantly to the model, as indicated by QSM (0.43, *P =* 0.003), in comparison to NfL blood levels (0.14, *P =* 0.71).

## Discussion

In this study adults with ADHD and healthy controls were investigated by QSM to assess magnetic abnormalities indicating locally increased iron deposition in the brain. In addition, blood‐serum levels of NfL were assessed as an indicator of CNS neuroaxonal vulnerability. QSM analysis revealed a distinct distribution of local susceptibility in ADHD patients and healthy volunteers. Statistically significant differences in iron content between controls and ADHD were found, with higher iron content in adult ADHD in the precentral cortex and several other brain structures. The observed statistical relationship between increased precentral cortex iron and elevated NfL blood levels may indicate increased neuroaxonal vulnerability in the adult ADHD group. To our knowledge this is the first study to investigate NfL, and the combination of QSM and NfL in ADHD.

Several longitudinal cohort‐studies have confirmed an association between adult ADHD and increased risk of dementia in old age.^3^, ^4^, ^5^ However, there is a lack of clinical studies investigating the mechanisms that might link ADHD in middle‐age to neurodegenerative dementia in old age. The current study aimed to provide insights into a possible relationship between increased brain iron content, as a proxy for oxidative stress, neuroaxonal vulnerability in the CNS, as reflected by increased plasma‐NfL, and adult ADHD. While oxidative stress has been implicated as an important underlying mechanism in several brain disorders, including neurodegenerative dementia,^26^, ^52^, ^53^ preliminary data suggest an inadequate response to oxidative stress in ADHD.^54^

In the current study, plasma NfL concentrations were measured using an ELLA automated microfluidic immunoassay platform. The fourth‐generation ELLA immunoassay platform has been shown to be comparable in accuracy to established advanced ELISA methods such as SiMoA.^39^ Several neuropsychiatric disorders are characterized by elevated blood NfL, which is considered to reflect neuroaxonal vulnerability and damage in the brain.^37^ In dementia and early stages of Alzheimer's disease, increased NfL is an established blood biomarker for neurodegenerative brain change.^40^, ^42^ In addition, associations between elevated NfL and reduced cognitive performance have been reported in several other neurological conditions.^55^ Interestingly, in Alzheimer's disease, elevated NfL has been suggested to be particularly associated with deficits in the cognitive subdomain of attention.^56^ Although we do not find a group difference between ADHD (diagnosis or symptom severity) and healthy controls in our study, these previous observations might nevertheless be consistent with our finding of a significant statistical association between elevated NfL and precentral brain iron load in adult ADHD. This may suggest that NfL is more relevant to individual iron‐related brain pathology than to the behavioral syndrome that defines the clinical diagnosis of ADHD. However, as the observed effect size was small, type‐2 error cannot be excluded.

We acknowledge the limitations of this study. The small sample size and the naturalistic medication regime in the ADHD‐group has made subgroup analyses of lifestyle and medication effects impossible. Although the control group was slightly younger and had a higher proportion of women, the differences were not statistically significant. However, the regional nature of the study population, which was recruited locally in Switzerland, warrants interregional follow‐up. Our current study data were obtained cross‐sectionally, which limits possible conclusions about trends over time. While our finding of an association between high QSM measures and increased serum NfL may be consistent with previous findings in Parkinson's Disease,^45^ further longitudinal studies are needed to investigate the possible relevance of our findings with respect to risk for neurodegenerative diseases. In addition, a follow‐up study with sufficient power for multivariate clustering and sophisticated prediction statistics could confirm interactions between NfL and other potentially modifiable risk factors. There is ongoing debate about the effects of long‐term psychostimulant medication on iron accumulation in the CNS of persons with ADHD.^57^, ^58^, ^59^ As various psychostimulants, such as cocaine and 3,4‐methylenedioxymethamphetamine (MDMA) can affect both brain iron accumulation,^60^ and NfL levels,^38^, ^61^ their regular use was an exclusion criterion in our study. However, as 59.4% of our study participants with ADHD were receiving medication with methylphenidate or dexamphetamine, a potential effect on measured brain iron and blood NfL levels cannot be excluded, and must be considered as a limitation of the present study. Furthermore, the QSM values obtained in the white matter regions of our cohort could also indicate individual variations in myelinization.^30^ However, follow‐up studies with specifically targeted neuroimaging strategies would be required to answer this question.^62^

Our QSM data show increased brain iron in the precentral cortex and several other brain structures in adults with ADHD. Results from very small regions, such as the supramarginal gyrus, must be interpreted with caution due to the increased risk of inaccuracies in parcellation and atlas mapping. QSM values in brain gray matter are considered to indicate Ferritin‐bound Fe^3+^, which may be related to Fenton‐oxygenation in neurodegenerative brain diseases.^15^, ^16^, ^63^ Our findings of increased brain iron in adults with ADHD may seem counterintuitive given previous observations of reduced brain iron in children with ADHD.^34^, ^35^, ^64^ Further studies are needed to replicate our findings, and to clarify whether increased brain iron in adults with ADHD may be an evolutionary physiological response to low brain iron in childhood, not taking into account today's longer life expectancy. It is also important to consider that due to the blood–brain barrier, iron uptake and iron metabolism are not identical inside and outside the CNS^65^ – and as a consequence, iron levels measured in the brain (as in our study) and peripheral iron levels (as in previous studies of iron in ADHD) may not directly correlate. The association observed in our study between increased susceptibility in the precentral cortex and increased NfL in adult ADHD suggests a potential link between excess brain iron and neuroaxonal vulnerability, possibly mediated by oxidative stress. To our knowledge this is the first report of a statistical relationship between increased brain iron and indicators of neuroaxonal damage in adult ADHD. Further clinical studies are needed to confirm the importance of midlife brain iron load for risk of dementia in old age, and a possible association with previously reported reduced brain iron in children with ADHD. This also applies to personalized prevention strategies aimed at reducing the risk of dementia in adults with ADHD by taking into account individual systemic iron turnover.

In conclusion, our QSM data show increased brain iron in the precentral cortex and several other brain structures of adults with ADHD. The observed association between increased precentral magnetic susceptibility and increased NfL suggests a potential link between local brain iron excess and neuroaxonal vulnerability, which may have implications for brain health in ADHD individuals as they age.

## Author contributions

Conceptualization: PU. Data curation: CS, PS. Formal analysis: JB, CS, SK. Funding acquisition: PU. Investigation: PU. Methodology: PU, JB, SK, PG. Project administration: PU. Resources: PU. Software: JB. Supervision: PU. Validation: PU, JB. Visualization: JB, SK. Roles/Writing – original draft: JB. Writing – review and editing: SK, CS, JH, FB, PG, PvZ, NP, XL, KL, BQ, PU, ES.

## Disclosure statement

All the authors declare no conflict‐of‐interest of financial benefit related to this manuscript.
